# Differential insular cortex sub-regional atrophy in neurodegenerative diseases: a systematic review and meta-analysis

**DOI:** 10.1007/s11682-019-00099-3

**Published:** 2019-04-23

**Authors:** Yasmine Y. Fathy, Susanne E. Hoogers, Henk W. Berendse, Ysbrand D. van der Werf, Pieter J. Visser, Frank J. de Jong, Wilma D.J. van de Berg

**Affiliations:** 1 Department of Anatomy and Neurosciences, Section Clinical Neuroanatomy and Biobanking, Amsterdam UMC, Vrije Universiteit Amsterdam, Amsterdam Neuroscience, De Boelelaan 1108, 1081 HZ Amsterdam, Netherlands; 2 Department of Neurology, Erasmus Medical Center, Postbus, 2040 3000 Rotterdam, CA Netherlands; 3 Department of Neurology, Amsterdam UMC, Vrije Universiteit Amsterdam, Amsterdam Neuroscience, De Boelelaan 1117, 1081 HZ Amsterdam, The Netherlands; 4 Department of Anatomy and Neurosciences, Section Neuropsychiatry, Amsterdam UMC, Vrije Universiteit Amsterdam, Amsterdam Neuroscience, De Boelelaan 1108, 1081 HZ Amsterdam, The Netherlands; 5 Department of Psychiatry and Neuropsychology, Maastricht University, Maastricht, Netherlands

**Keywords:** Insular cortex, Parkinson’s disease, Frontotemporal dementia, Alzheimer’s disease, Voxel based morphometry, Cognition, Emotion, Perception, Anatomic likelihood estimation, Neurodegeneration

## Abstract

The insular cortex is proposed to function as a central brain hub characterized by wide-spread connections and diverse functional roles. As a result, its centrality in the brain confers high metabolic demands predisposing it to dysfunction in disease. However, the functional profile and vulnerability to degeneration varies across the insular sub-regions. The aim of this systematic review and meta-analysis is to summarize and quantitatively analyze the relationship between insular cortex sub-regional atrophy, studied by voxel based morphometry, with cognitive and neuropsychiatric deficits in frontotemporal dementia (FTD), Alzheimer’s disease (AD), Parkinson’s disease (PD), and dementia with Lewy bodies (DLB). We systematically searched through Pubmed and Embase and identified 519 studies that fit our criteria. A total of 41 studies (*n* = 2261 subjects) fulfilled the inclusion criteria for the meta-analysis. The peak insular coordinates were pooled and analyzed using Anatomic Likelihood Estimation. Our results showed greater left anterior insular cortex atrophy in FTD whereas the right anterior dorsal insular cortex showed larger clusters of atrophy in AD and PD/DLB. Yet contrast analyses did not reveal significant differences between disease groups. Functional analysis showed that left anterior insular cortex atrophy is associated with speech, emotion, and affective-cognitive deficits, and right dorsal atrophy with perception and cognitive deficits. In conclusion, insular sub-regional atrophy, particularly the anterior dorsal region, may contribute to cognitive and neuropsychiatric deficits in neurodegeneration. Our results support anterior insular cortex vulnerability and convey the differential involvement of the insular sub-regions in functional deficits in neurodegenerative diseases.

**Electronic supplementary material:**

The online version of this article (10.1007/s11682-019-00099-3) contains supplementary material, which is available to authorized users.

## Introduction

The insular cortex generally sub-serves the integration of autonomic, viscero-sensory, and interoceptive functions and plays a role in cognition, decision-making and processing of emotions (Augustine 1996; Flynn 1999; Christopher et al. 2014; Uddin et al. 2017). Through specialized cells in the anterior insula called von Economo neurons (VENs), it is hypothesized to play a role in social awareness and consciousness (Craig 2009; Allman et al. 2011; Evrard et al. 2012). The diversity of functions played by the insular cortex is paralleled to its widespread connectivity with functionally different brain regions. The anterior ventral insula has preferential connections to limbic regions and is involved in emotional processing and consciousness. Whereas, the posterior insula has connections with neocortical somatosensory regions, and plays a role in interoception. In addition, the anterior insular cortex encompasses a transitional dorsal area connected to multiple limbic and neocortical brain regions, which plays a role in cognition and decision making (Mesulam and Mufson 1982; Augustine 1996; Uddin et al. 2017).

The insular cortex has shown grey matter abnormalities across 26 neurodegenerative and neuropsychiatric diseases (Crossley et al. 2014). Its atrophy may contribute to an array of cognitive and neuropsychiatric deficits depending on the sub-regions involved. In frontotemporal dementia (FTD), a family of disorders with behavioral, socio-emotional, and language deficits, the anterior part of the insula appears to be selectively vulnerable to degeneration (Seeley 2010). Moreover, a multi-center study showed that insular cortex atrophy contributed to a high diagnostic accuracy, using MRI, in diagnosing the behavioral variant of FTD (Meyer et al. 2017). Similarly, atrophy of the insular cortex is associated with the development of neuropsychiatric deficits such as agitation, apathy, and psychosis in patients suffering from Alzheimer’s disease (AD) (Rosenberg et al. 2015). In Parkinson’s disease (PD), voxel based morphometry (VBM) studies reported insular cortex atrophy particularly in PD patients with mild cognitive impairment (MCI) compared to patients without MCI. Insular cortex atrophy also correlated with executive dysfunction in the PD-MCI group (Lee et al. 2013; Mak et al. 2014). Moreover, in Dementia with Lewy bodies (DLB), an entity similar to PD with early onset dementia, the anterior insula was shown to be atrophic in patients during the prodromal phase of the disease (Blanc et al. 2016). Data on the insula’s dense connections, richness of interconnections, central position in networks, and variety of functions indicate that it plays a central role in the brain as a structural hub (van den Heuvel and Sporns 2013). However, the precise involvement of the insular sub-regions in neurodegenerative diseases and their associated functional deficits remain as yet undefined.

VBM, used in the analysis of in vivo structural imaging, has gained much popularity due to its relative user-friendliness and its capacity to reveal changes in brain volumes as indicators of neurodegeneration (Ashburner and Friston 2000). VBM assesses changes in whole brain volumes using a voxel-by-voxel comparison of grey or white matter between groups through parametric tests and a user set *p* value (Whitwell 2009). It is often difficult, however, to compare across individual studies using VBM, due to limited sample sizes, the use of different corrections, registration and pre-processing tools (Whitwell 2009). This could in turn lead to false positive and negative results as well as inconsistent findings, hence limiting the reliability of individual studies. Therefore, in this study, we used a meta-analytic approach across multiple studies to quantitatively identify the insular sub-regions that consistently showed significant differences across disease groups.

Considering the heterogeneity of the insular cortex and its potential contribution to a myriad of functions affected in neurodegenerative diseases, we aimed to systematically collect and quantitatively analyze data on insular cortex sub-regional atrophy in neurodegenerative disorders. We hypothesized that selective atrophy of the anterior insular cortex would be associated with cognitive and neuropsychiatric deficits in neurodegenerative diseases. To address this issue, we studied insular cortex sub-regional atrophy and its contribution to cognitive and neuropsychiatric deficits across FTD, AD, PD and DLB using Anatomic Likelihood Estimation (ALE) as a meta-analytic quantitative approach.

## Methods

### Systematic search

This systematic review is reported according to the Preferred Reporting Items for Systematic Reviews and Meta-Analyses (PRISMA) (Moher et al. 2010). Search syntaxes were tailored to retrieve VBM studies assessing the role of the insular cortex in AD, FTD, PD, and DLB using two bibliographic databases: PubMed and Embase. Search syntaxes included a combination of the following: (“insula” OR “von Economo neurons”) AND (Parkinson’s OR Dementia Lewy body OR frontotemporal OR Alzheimer’s) AND (imaging OR networks OR voxel based morphometry) AND (cognitive impairment OR dementia OR affective OR behavioral OR neuropsychiatry) as well as their appropriate Medical Subject Headings (MeSH). The search was run in January 2017. Articles were then screened using a predefined eligibility criteria: 1) English language 2) includes human subjects only 3) Peer-reviewed studies 4) Primary studies only 5) Studies on any of the four diseases mentioned including the FTD disorders such as semantic dementia, behavioral variant FTD (bvFTD), and progressive aphasias. Studies were included regardless of the presence of a healthy control group. For the meta-analysis, studies were included if they reported the insular coordinates in Talairach or MNI space. The stereotactic coordinates of the insular cortex were included from whole-brain analyses. Authors were contacted, when necessary, to retrieve the exact insular coordinates or in case of missing information. When coordinates were not retrieved, studies were excluded from the meta-analysis. References of the included publications were screened for additional relevant studies.

Following a similar meta-analytic approach, studies were then categorized into 6 main functional domains: cognition, speech, emotion, perception, behavior, and affective-cognition. Studies that assessed an emotion or complex emotional states such as apathy were categorized in the emotional domain. Similarly, studies that assessed a cognitive function were added to the cognitive domain. The affective-cognition category included functions with both emotional and cognitive aspects such as empathy, the recognition of an emotion or enhancement of cognition through emotion. Categories were defined based on the type of functions retrieved as performed in a similar study (Criaud et al. 2016).

**Fig. 1:**
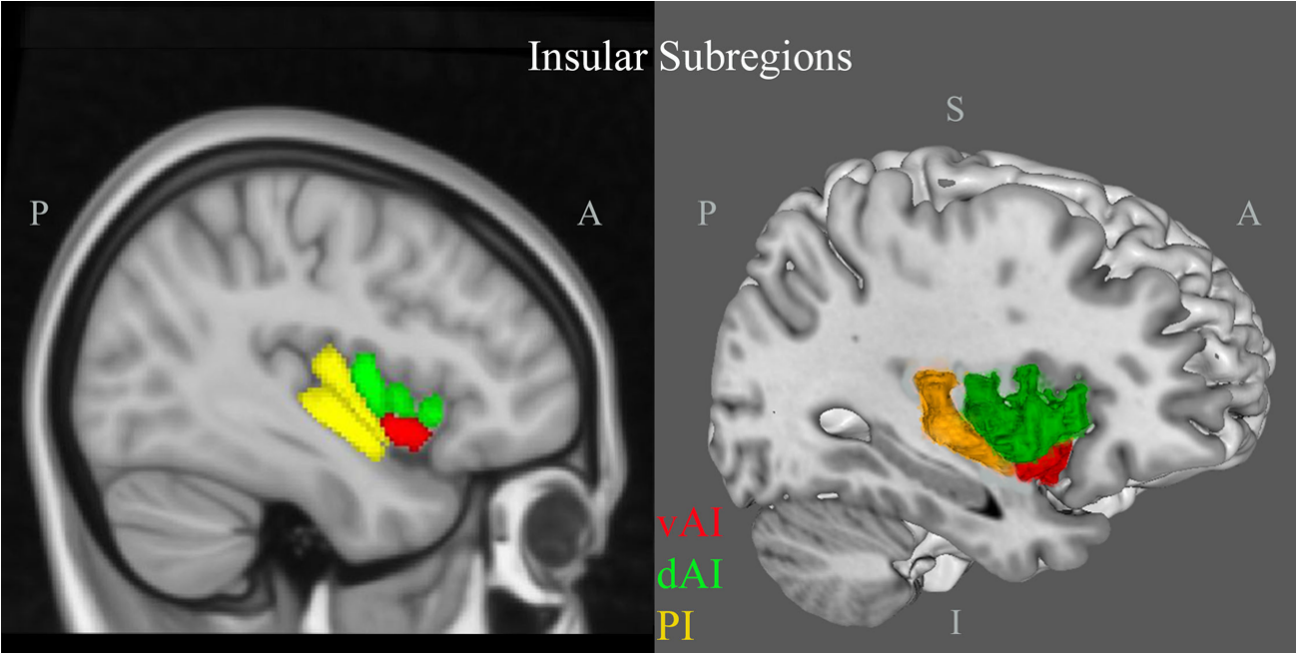
Insular Cortex Sub-regions in MRI. The central sulcus generally divides the insular cortex into anterior and posterior subdivisions. The anterior insular cortex is further divided into ventral (red) and dorsal sub-regions (green) and the dorsal insula can be divided into an anterior and mid-region. The left figure shows the sub-regions of the insular cortex based on the Hammers_mith probabilistic atlas (Faillenot et al., 2017). Surface rendering of the insular sub-regions in 3D is shown on the right. A: anterior, dAI: dorsal anterior insula, I: inferior, P: posterior, PI: posterior insula, S: superior, vAI: ventral anterior insula

Meta-analysis included assessing 1) insular cortex atrophy per disease group and across all groups combined: FTD, AD, PD, and DLB, and 2) assessment of functional domains and their relationship with atrophy in patients compared to controls. A whole-group analysis was performed to define convergence clusters of atrophy in the insular cortex across all diseases together. Each disease group was then assessed separately, while PD and DLB studies were combined (PD/DLB group) due to the small number of DLB studies as well as similarities between the two diseases. To compare insular contribution to each disease, we performed contrast studies: AD versus FTD, AD versus PD/DLB, and FTD versus PD/DLB. Subsequently, conjunction maps were plotted to identify overlapping insular sub-regions of atrophy across the three defined disease groups. Meanwhile, we assessed the cognitive and psychiatric correlates, categorized by functional domain, of atrophy in the insular cortex across the above-mentioned neurodegenerative diseases. Only studies that assessed a correlation between atrophy and a functional domain, regardless of the presence of control groups, were included in the meta-analysis. Peak insular coordinates derived from correlation between VBM atrophy and function were included. An anatomical map with central sulcus as a landmark was used to separate between the anterior and posterior insular divisions. The anterior insula was divided into ventral and dorsal sub-regions based on the macro- and microscopic landmarks as previously studied (Naidich et al. 2004; Morel et al. 2013) (Fig. 1).

### Statistical analysis

Analysis of the VBM data was performed using an Anatomic Estimation Likelihood (ALE) approach, using GingerALE 2.3.5 (http://brainmap.org/ale/). This meta-analytic technique uses the coordinates of studies in which significant differences were observed and displays them as 3D Gaussian probability distributions (Laird et al. 2005). The MNI coordinates of insular foci were extracted from each study and converted to Talairach using Lancaster transform applied in GingerALE (Eickhoff et al. 2009).

Statistical significance in single studies was determined using an uncorrected *p* < 0.001 and cluster size 200mm^3^. Other available approaches for the computation of convergence of anatomic probabilities existing above chance include False discovery rate correction (FDR), Family wise error correction (FWE), and cluster level analysis, which are commonly used at a *p* value <0.05. The latter approach, although more sensitive than FDR and FWE and protects from false positives, may not be suitable for VBM studies as previously mentioned (Ashburner and Friston 2000; Eickhoff et al. 2012). Moreover, as the FDR and FWE approaches are more conservative, they would yield very restricted results (Eickhoff et al. 2012). An uncorrected p was used in this analysis as a quick and deterministic analytical method since only insular cortex rather than whole-brain coordinates were used. Contrast analyses used the same parameters as well as permutation testing with 10,000 iterations. Clusters of atrophy in the insula from different studies were then mapped on a Colin-27 template in Talairach space using Mango software for image processing (www.ric.uthscsa.edu/mango).

## Results

### Systematic search

**Fig. 2:**
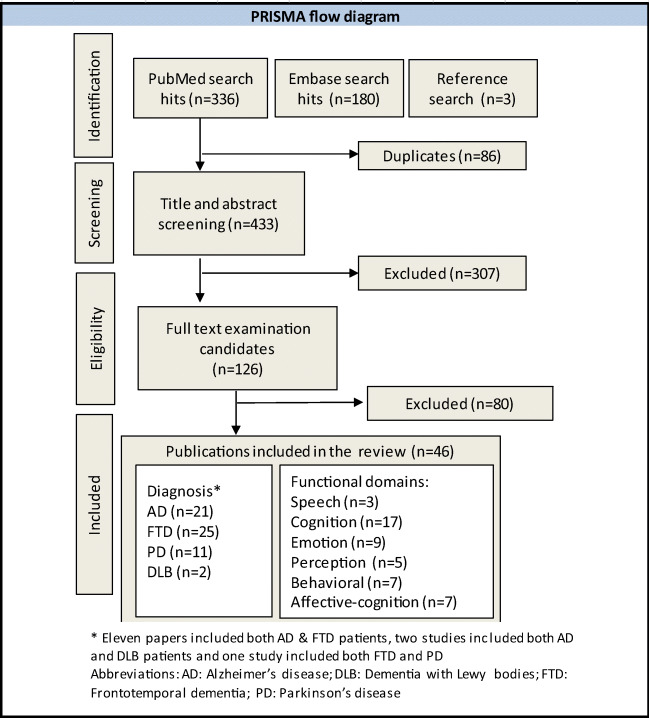
PRISMA flow Diagram of study selection. Diagram shows the search results, removal of duplicates, and final articles included after title and abstract screening and full text examination. A total of 46 studies fitting the inclusion criteria were added. The functional categories assessed in meta-analysis include speech, cognition, emotion, perception, affective-cognition, and behavior

The systematic search through Embase and Pubmed yielded 519 studies. After title and abstract screening and removal of duplicates, 159 studies qualified for full text examination. A total of 46 original articles fulfilled the inclusion criteria and were included in this review. For the meta-analysis, the stereotactic insular coordinates of atrophy were retrieved from a total of 41 studies fulfilling the eligibility criteria. The total number of subjects in the included studies is 2261. There are 25 studies on FTD, 21 on AD (including 11 studies comparing FTD and AD), 11 studies on PD, and 2 on DLB (both comparing DLB and AD). Figure 2 shows a detailed flow diagram of the study selection process.

The cognitive domain included studies on cognitive impairment assessed by global cognitive tests (*n* = 7), theory of mind, assessing the ability to place oneself in other’s minds (*n* = 2), and self-awareness (*n* = 1). The emotion domain included studies on apathy (*n* = 4), happiness (n = 1), music aversion (n = 1), fear conditioning and emotional blunting (n = 1). The affective-cognitive domain contained studies on recognition of emotions and emotional enhancement of cognition (*n* = 6). The perception domain included delusions (n = 2) and hallucinations (*n* = 3). Behavior contained studies on aberrant eating (n = 4), disgust behavior (n = 1), pathological gambling (n = 1), auditory hedonic behavior (n = 1) and prosocial behavior (n = 1). The domain speech included aphasia, verbal agility, and speech fluency (n = 3) (Supplementary Table 1). There were 2 studies on pain, temperature, and blood pressure. Two further studies reported a relationship between the insula and activities of daily living. The latter studies were not included in the meta-analysis due to the heterogeneity of functions. In the meta-analysis, peak insular coordinates were pooled and analyzed if they corresponded to any of the following domains: cognition, emotion, perception, speech, affective-cognition, and behavior (Table 1).

**Table 1:** Characteristics of VBM studies

Study	Diagnosis	Sample size	Mean age/Group	Insular region Atrophy	Functional domain	Correlation Volume/Atrophy vs function	p value GM Multiple comparison	MRI Field strength	Software	FWHM
(Li et al. 2016)	AD	AD = 21 HC = 25	AD = 68.19 ± 9.07 HC = 64.52 ± 6.44	Bilateral insula	Affective-cognition: Impaired recognition of emotional images-emotional memory (EM)	Positive: volume of bilateral insula and EM	p < 0.05 FWE corrected	3.0 T	SPM8	8 mm
(Amanzio et al. 2016)	bvFTD	FTD = 15 HC = 15	bvFTD =68.65 ± 8.68 HC = 62.0 ± 4.4	Left insula	Activity Instrumental Activities of daily living (iADL)	Positive: Left insula volume and iADL scores	*p* < 0.005 corrected	1.5 T	SPM8	8 mm
(Alzahrani et al. 2016)	PD	PD = 65 HC = 24	PD = 67.1 HC = 62.79 (9.77)	Left insula	Emotion: Apathy	Negative: Left insula volume and apathy	p < 0.001 corrected	1.5 T	SPM8	8 mm
(Dermody et al. 2016)	FTD AD	bvFTD =24 AD = 25 HC = 22	bvFTD =63.0 AD = 66.1(8.0) HC = 68.2(6.7)	Left insula	Affective-Cognitive: Empathy	Positive: Left insula volume and empathy in bvFTD	p < 0.001 uncorrected	3 T	FSL	8 mm
(Blanc et al. 2016)	Pro-DLB and pro-AD	Patients =55 HC = 33	Pro-DLB =67.5 Pro-AD 69.3= HC = 72.4 ± 10.4	Bilateral insula	Cognitive: MCI	No correlation assessed.Bilateral insular atrophy and pro-DLB diagnosis	p < 0.05 FWE corrected	3 T	SPM8	8 mm
(Mandelli et al. 2016)	FTD: nfvPPA and bvFTD	FTD = 48 HC = 34	FTD = 64.8 HC = 62.3	1.nfvPPA: Left SPGI 2.bvFTD: bilateral insula	1-Speech: verbal agility (nfvPPA) 2-Behavior: Aberrant eating (bvFTD)	Positive: 1-Left SPGI and verbal agility in nfvPPA Negative 2- bvFTD: Bilateral VA insulae volume and aberrant eating	p < 0.05 FWE corrected	1.5/3 T	SPM8	8 mm
(Heitz et al. 2016)	DLB and AD	Patients = 48 HC = 16	Patients =68.9 HC = 68.3 ± 10.5	Left insula	Cognitive & affective-cognition: Theory of mind, faux pas recognition (social sensitivity)	Positive: Left insula atrophy and Theory of mind deficits in DLB	P < 0.05	3 T	SPM12	8 mm
(Chen et al. 2016)	PD	Patients = 37 HC = 21	Patients =61.9 HC = 61.95 ± 5.40	Bilateral insula	Cognition & Disease severity	Positive: right insula atrophy & disease duration,UPDRS score -No correlation with cognition	*p* < 0.01 alphasim correction *p* < 0.05 Bonferroni correction	3 T	SPM8	8 mm
(O'Callaghan et al. 2016)	FTD	Patients = 22 HC = 22	Patients =64.8 ± 8.8 HC = 64.8 ± 11.1	Left anterior insula	Behavior: Pro-social behavior	Positive: left anterior insula volume & Prosocial behavior	p < 0.05 FWE corrected	3 T	FSL	8 mm
(Zhang et al. 2015)	PD	PD = 35 HC = 20	PD = 61.86 ± 8.98 HC = 59.36 ± 6.36	PD-MCI: Left insula	Cognition: Memory impairment	No correlation assessed. PD-MCI and atrophy in the left insula	p < 0.05	3 T	SPM5	8 mm
(Fletcher et al. 2015b)	FTD and AD	FTD = 56 AD = 17 HC = 50	Patients = 64.7 HC = 67.5	1.Bilateral Mid- posterior insula	Behavior: Music aversion	Positive: Atrophy and music aversion in FTD	p < 0.05 FWE corrected	3 T	SPM8	6 mm
(Fletcher et al. 2015a)	FTD and PD	Patients = 78 HC = 20	Patients =64.95 HC = 67.5	Right mid and posterior insula	Autonomic: Pain & temperature	Positive: Atrophy and pain and temperature changes in FTD	p < 0.05	3 T	SPM8	6 mm
(Hu et al. 2015)	MCI and AD	Patients = 293 HC = 131	Patients =74.6 ± 7.5 HC = 75.6 ± 5.0	Left Insula	Emotion: Agitation	Positive: Atrophy and agitation in combined group MCI and AD	*P* < 0.05 FWE corrected	1.5 T	SPM8	8 mm
(Sturm et al. 2015)	FTD	FTD = 96 HC = 34	FTD = 61.9 ± 7.3 HC = 64.9 ± 9.3	Left anterior insula	Emotion: Happiness	Positive: Atrophy & higher happiness behavior	p < 0.05 FWE corrected	1.5/3 T	SPM5	8 mm
(Ting et al. 2015)	MCI/early AD (delusional vs non-delusional)	Patients = 58	Patients =74.4	Right Insula	Perception: Delusions	Positive: Insula atrophy and delusions	p < 0.001 uncorrected p < 0.05 FWE and FDR corrected	1.5 T	SPM8	10 mm
(Woolley et al. 2015)	AD, FTD	Patients = 305 FM = 25 HC = 90	*Patients =61.8 FM = 48.2 ± 12.4 HC(69.4 ± 7.0)	Bilateral Ventro-anterior Insula	Behavior & affective- cognition: 1-Disgusting behavior 2- Disgust Recognition	Positive: Bilateral anterior insula atrophy and disgust behavior/ recognition in FTD	1-p < 0.05 FWE 2-p < 0.005 FWE uncorrected	1.5,3,4 T	SPM5	8 mm
(Blanc et al. 2014)	AD	Patients = 39 HC = 39	Patients =76.2 HC = 78.8	Right anterior insula	Perception: Hallucinations	Positive: insula atrophy and hallucinations	p < 0.001 uncorrected p < 0.05 FWE corrected	1.5 T	SPM12b	8 mm
(Cerasa et al. 2014)	PD	Patients = 24 HC = 24	Patients =58.65 HC = 60.3 ± 9.1	Right insula	Behavioral: Pathological Gambling	No correlation between insula atrophy and test for gambling	p < *0.05* FWE corrected	3 T	SPM8	10 mm
(Kumfor et al. 2014)	FTD and AD	Patients = 27 HC = 12	Patients =67.8 HC = 71.3 ± 5.0	Right Insula	Affective-cognition: Emotional enhancement of memory	Positive: Emotional enhancement of memory and integrity of right insula in FTD	p < 0.005 uncorrected	3 T	FSL	8 mm
(Lee et al. 2014)	PD-MCI	PD = 51 HC = 25	PD = 71.36 HC = 70.0 ± 3.4	Left insula-	Cognitive: Frontal executive functions	No correlation between insula atrophy and executive functions	p < 0.001 uncorrected	3 T	SPM8	6 mm
(Gama et al. 2014)	PD	PD = 39 HC = 10	PD = 67.1 ± 8.4 HC = 68.1 ± 7.0	Left insula	Perception: Visual hallucinations	Positive: Atrophy of left insula and hallucinations	P < 0.05	1.5 T	SPM8	12 mm
(Mak et al. 2014)	PD- MCI vs no MCI	Patients = 90	Patients = 64.95 ± 7.54	Left insula	Cognitive: Executive function & attention	Negative: Left insula atrophy and executive function/ attention	p < 0.001 uncorrected p < 0.05	3 T	SPM8	8 mm
(Perry et al. 2014)	bvFTD	FTD = 91	FTD = 59.7 ± 8.4	Right anterior insula	Behavior: Aberrant Eating and sweet preference	Positive: Insula volume and aberrant eating	*P* < 0.05 FWE corrected	1.5/ 3/ 4 T	SPM8	8 mm
(Shany-Ur et al. 2014)	AD and FTD	Patients = 78 HC = 46	Patients = 62.1 HC = 69.9 ± 7.1	Right anterior and posterior insula	Cognitive: Self-awareness	Positive: 1- Right anterior insula & awareness of ADLs, cognitive abilities, and interpersonal abilities. 2- Bilateral insular atrophy & awareness of emotional control	P < 0.05 FWE corrected *P* < 0.001 uncorrected	1.5/3/4 T	SPM5	–
(Shine et al. 2014)	PD (hallucinators vs non-hallucinators)	Patients = 22	Patients = 63.21	Bilateral anterior insula	Perception: hallucinations	Positive: Bilateral anterior insula atrophy and BPP score (hallucinations)	P < 0.05 FDR corrected	3 T	SPM8	8 mm
(Cerami et al. 2014)	bvFTD	FTD = 14HC = 20	FTD = 63.4 ± 7.47 HC = 62.8 ± 7.9	Left posterior insula	Emotion: Emotional attribution of empathy	Positive: Left insula atrophy and emotion attribution	p < 0.05 FWE corrected	3.0 T	SPM8	8 mm
(Couto et al. 2013)	PNFA and bvFTD	Patients =22 HC = 18	Patients = 67.57 HC = 69.8 ± 7.3	1-Bilateral insula- both groups 2- bilateral anterior insula- bvFTD	1- Affective-Cognition: Face recognition, emotion recognition, 2- Cognition: theory of mind	Negative1-bilateral insula atrophy & Face recognition in PNFA 2- Emotion & bilateral insula atrophy 3-TOM: bilateral insula atrophy in PNFA	p < 0.05	1.5 T	SPM8	12 mm
(Stanton et al. 2013)	AD and PSP	Patients =17	Patients = 72.68	Left insula	Emotion: Apathy Emotional blunting	Positive: Left insular atrophy & emotional blunting and apathy	p < 0.05	3 T	SPM5	8 mm
(Kumfor et al. 2013)	FTD (bvFTD, SD, PNFA)	Patients =40 HC =27	Patients = 63.69 HC = 64.3 ± 3.7	Disgust recognition: left ventral anterior insula	Affective-Cognition: Negative Emotion recognition	Positive: Left ventral anterior insula volume with disgust recognition in bvFTD and SD	p < 0.05 FWE corrected	3 T	FSL	8 mm
(Lee et al. 2013)	PD ± dementia	Patients =32 HC = 16	Patients = 69.1 HC = 69.5 ± 6.3	Anterior insula: Short insular gyrus	Cognition: Dementia	Decreased anterior insula volume in PDD	p < 0.001 uncorrected	1.5 T	SPM2	8 mm
(Nakaaki et al. 2013)	AD (delusional vs non-delusional)	Patients = 53	Patients = 76.94	Left insula	Perception: Delusions	Positive: Left insular atrophy & delusions	p < 0.05 FDR corrected	1.5 T	SPM5	12 mm
(Eslinger et al. 2012)	FTD(bvFTD, PNFA, SD)	Patients = 26 HC = 16	FTD = 68.45 HC = 75.0 ± 6.6	Left anterior insula	Emotion: Apathy	Negative: Left anterior insular volume & apathy evaluation scale in bvFTD	*p* < 0.0001 uncorrected *p* < 0.025 uncorrected	3 T	SPM99	12 mm
(Hsieh et al. 2012)	SD-FTD AD	FTD = 9 AD = 12 HC = 15	FTD = 62.6 ± 5.4 AD = 62.9 ± 8.2 HC = 64.2 ± 6.4	Bilateral insula	Affective-Cognitive: Emotion Recognition from faces and music	Positive: Insula volume and emotion recognition	p < 0.001 uncorrected	3 T	FSL	8 mm
(Vasconcelos et al. 2011)	Mild AD	Patients = 19	Patients = 75.2 ± 4.7	Right anterior insula	Cognition: Global (MMSE) and disability assessment for dementia	Positive: Right insular atrophy & disability assessment for dementia scores and MMSE	P < 0.001 uncorrected	1.5 T	SPM5	8 mm
(Omar et al. 2011)	FTD	Patients = 26 HC = 21	Patients = 63.81 HC = 67.0 ± 8.8	Bilateral anterior insula	Affective-cognition: Emotion recognition	Positive: Anterior insula atrophy and impaired emotion recognition from music and faces	p < 0.05 FDR corrected	1.5 T	SPM2	8 mm
(Song et al. 2011)	PD (MCI vs PDD)	Patients = 68	Patients = 70.76	left insula (PD-MCI)& right insula (PDD)	Cognitive: MCI and dementia	Positive: cognitive impairment and insular atrophy	p < 0.05	3 T	SPM8	6 mm
(Hu et al. 2010)	LPA and PNFA (AD & FTD)	†Patients = 23	Patients = 63.89	left insula	Speech: aphasia	Positive: Left insula atrophy & aphasia in FTD	p < 0.05 FWE corrected	3 T	SPM5	–
(Reijnders et al. 2010)	PD	Patients = 60	Patients = 62.0 ± 10.1	Bilateral insula	Emotion: Apathy	Positive: Bilateral insula atrophy & apathy scores	p < 0.05 FDR corrected	3 T	SPM8	10 mm
(Ash et al. 2009)	FTD (PNFA, SD, Soc/Exec)	†Patients = 22 HC = 10	Patients = 67.34 HC = 69.5 ± 5.1	Left insula	Speech: speech fluency	Positive: Left atrophy and fluency in PNFA & SD	p < 0.001	1.5/3 T	SPM5	8 mm
(Kipps et al. 2009)	FTD AD	FTD = 21 HC = 12	FTD = 62.1 ± 6.6 HC = 66.4 ± 4.9	Left insula	Affective-cognition: Emotion Recognition	No correlation assessed	P < 0.05 FDR	3 T	SPM5	8 mm
(Hoefer et al. 2008)	AD & FTD	†Patients = 37 HC = 17	Patients = 62.61 HC = 66.7 ± 8.6	Left insula	Emotion: Fear conditioning & emotional blunting	Positive: Left insular volume & reactivity to unconditioned stimulus in FTD	p < 0.05 FWE corrected	1.5 T	SPM2	12 mm
(Seeley et al. 2008)	FTD	Patients =45 HC = 45	Patients = 64.16 HC = 68.3 ± 7.9	Bilateral Anterior and posterior insula	Cognitive: CDR	No correlation assessed: low CDR & anterior insula atrophy. high CDR & bilateral posterior insula	p < 0.05 FWE corrected	1.5 T	SPM2	12 mm
(Woolley et al. 2007)	FTD, AD, SD	†Patients = 27 HC = 18	Patients = 59.5 HC = (57.2 ± 8.1)	Right anterior insula	Behavior: Binge Eating	Positive: Binge eating and right anterior insula atrophy	p < 0.05 corrected	1.5 T	SPM2	12 mm
(Farrow et al. 2007)	Early AD	Patients =7 HC = 11	Patients = 77 ± 7 HC = 70 ± 4	Bilateral insula	Cognitive: ADAS-TES performance	Positive: Left insula volume and ADAS-TES score	p < 0.05	1.5 T	SPM2	8 mm
(Whitwell et al. 2007)	FTD	Patients =16 HC = 9	Patients = 62.9 ± 7.6 HC = 62.6 ± 15.1	Right anterior insula	Behavior: Abnormal eating behavior	Positive: Pathological sweet tooth & right anterior insula atrophy	p < 0.05 corrected	1.5 T	SPM99	12 mm
(Rosen et al. 2005)	FTD, SD, PNFA& AD	Patients = 148	Patients = 64.8 ± 9.4	Anterior insula	Behavior: Apathy, eating disorders and aberrant motor behavior	Positive: Anterior insula atrophy and all behaviors. No correlation with specific function	p < 0.05 FWE corrected	1.5 T	SPM	12 mm

Characteristics of studies included in systematic review ane meta-analysis. All studies assessed and their corresponding subject demographics, insular atrophy and relationship with functional deficit, as well as technical details related to MRI and VBM are shown. *ADAS* Alzheimer’s disease assessment scale, *AD* Alzheimer’s disease, *ADL* activities of daily living *BPP* Bistable percept paradigm *bvFTD* behavioral variant FTD, *CDR* Clinical Dementia Rating scale, *DLB* Dementia with Lewy bodies, *EM* emotional memory, *FDR* False Discovery Rate, ***FM*** family member, *FTD* frontotemporal dementia, *FWE* family wise error, *FWHM* full width half maximum, *HC* healthy controls, ***LPA*** logopenic progressive aphasia, *MCI* mild cognitive impairment, *MMSE* mini-mental status examination, *nfvPPA* non-fluent variant primary progressive aphasia, *PD* Parkinson’s disease, *PDD* Parkinson’s disease with dementia, *PNFA* Progressive non-fluent aphasia, pro  Prodromal, *PSP* progressive supranuclear palsy, *SD* semantic dementia, *Soc/Exec* FTD subjects with social/executive deficits, *SPGI* superior precentral region of the dorsal anterior insula, *T* Tesla, *TOM* Theory of mind test; *VBM* voxel based morphometry

†Subjects that had an MRI (not total number of subjects)

### Literature review

#### Insular atrophy associated deficits in FTD

Studies on FTD showed a positive correlation between left insular atrophy and speech deficits such as verbal agility and verbal fluency, defined by words per minute (Ash et al. 2009; Mandelli et al. 2016). Insular atrophy was also found in FTD patients suffering from aphasia when compared with AD patients (Hu et al. 2010). Other studies found a positive correlation between insular atrophy and fear conditioning deficits, happiness, empathy deficits, and deficits in the recognition of emotion or enhancement of memory through emotion (Hoefer et al. 2008; Omar et al. 2011; Hsieh et al. 2012; Couto et al. 2013; Kumfor et al. 2013, 2014; Cerami et al. 2014; Sturm et al. 2015; Dermody et al. 2016). Kipps et al. reported that bvFTD patients with significant insular atrophy performed worse in recognizing negative emotions compared to positive ones, when compared with AD, controls, and bvFTD without significant insular atrophy (Kipps et al. 2009). Insular atrophy also positively correlated with behavioral deficits such as disgust, disinhibition, aberrant eating behavior, and compliance to social norms (Woolley et al. 2007; Whitwell et al. 2007; Perry et al. 2014; Woolley et al. 2015; O'Callaghan et al. 2016). One study showed a positive relationship between impaired activities of daily living in FTD with insular atrophy (Amanzio et al. 2016). Fletcher et al. reported a positive association between right middle and posterior insular atrophy and altered pain and temperature responsiveness while Sturm et al. reported higher cardiovascular activity and happiness with left anterior insular atrophy in FTD (Fletcher et al. 2015a; Sturm et al. 2015). Seeley et al. showed that the anterior insula was atrophic in FTD patients with low clinical dementia rating scale (CDR) scores and posterior insular atrophy in patients with higher CDR scores and thus, more cognitive impairment (Seeley et al. 2008). Left anterior insular atrophy also positively correlated with apathy in bvFTD (Eslinger et al. 2012). Rosen et al. showed that right anterior insular atrophy correlated with apathy, disinhibition, eating disorders, and aberrant motor behavior in FTD/semantic dementia (Rosen et al. 2005).

#### Cognitive and neuropsychiatric deficits in AD and PD

In AD studies, impairment in self-awareness and overestimation of one’s functions positively correlated with right anterior insular atrophy (Shany-Ur et al. 2014). General cognitive performance, assessed by Alzheimer’s disease assessment scale (ADAS), also positively correlated with insular atrophy (Farrow et al. 2007). Moreover, psychosis, including hallucinations and delusions, positively correlated with insular atrophy (Blanc et al. 2014; Ting et al. 2015). One study found a positive correlation between deficits in the recognition of emotions and bilateral insular atrophy (Li et al. 2016). Hu et al. found a positive correlation between agitation and insular atrophy in a cohort of MCI and AD (Hu et al. 2015). Disability in activities of daily living were assessed using Disability assessment for Dementia and showed a positive correlation with insular atrophy (Vasconcelos et al. 2011). In AD, the emotional component of apathy, emotional blunting, rather than the behavioral was associated with left insular atrophy (Stanton et al. 2013). While in PD, apathy correlated with insular atrophy as well as executive dysfunction and cognitive deficits (Reijnders et al. 2010; Alzahrani et al. 2016). Cognitive impairment studies in PD, however, showed variable results. Two studies showed insular atrophy in PD-MCI and PD dementia patient groups; while Lee et al. showed similar results as well as a correlation between insular atrophy and MMSE scores (Song et al. 2011; Lee et al. 2013; Zhang et al. 2015). Similarly, Mak et al. reported insular atrophy in PD and a negative correlation with executive functions (Mak et al. 2014). However, Lee JE et al. showed insular atrophy was present in PD with cognitive impairment but no correlation with executive functions (Lee et al. 2014). Chen et al. found bilateral insular atrophy in PD with normal cognition compared to controls but did not find a correlation with cognitive performance. On the other hand, the authors found a positive correlation between right insular atrophy and UPDRS III scores, indicative of a relationship with motor performance and disease progression (Chen et al. 2016). Gamma et al. reported left insular atrophy in PD patients with visual hallucinations but not cognitive impairment and Shine et al. reported bilateral anterior insular atrophy in correlation with bistable percept paradigm scores for the assessment of visual hallucinations in PD (Gama et al. 2014; Shine et al. 2014). One study assessed pathological gambling in PD but did not find a correlation with insular atrophy (Cerasa et al. 2014). Whereas in DLB, Blanc et al. reported bilateral insular atrophy in patients with MCI in the prodromal phase of the disease, while Heitz et al. found theory of mind deficits associated with attributing mental states to one-self and others, in correlation with insular atrophy in DLB patients (Blanc et al. 2016; Heitz et al. 2016).

#### Summary of deficits related to insular atrophy in neurodegeneration

Results from VBM studies show that insular cortex atrophy is related to functional deficits in all four diseases. In FTD, insular cortex atrophy was related to speech and language deficits, emotional, affective-cognitive deficits, as well several behavioral deficits. In AD and PD/DLB, insular cortex atrophy was mostly related to psychosis and cognitive impairment. Although the regional involvement of the insula has been variable across studies, the anterior insula has been implicated in multiple deficits.

### Meta-analysis

#### Disease group analysis

**Fig. 3:**
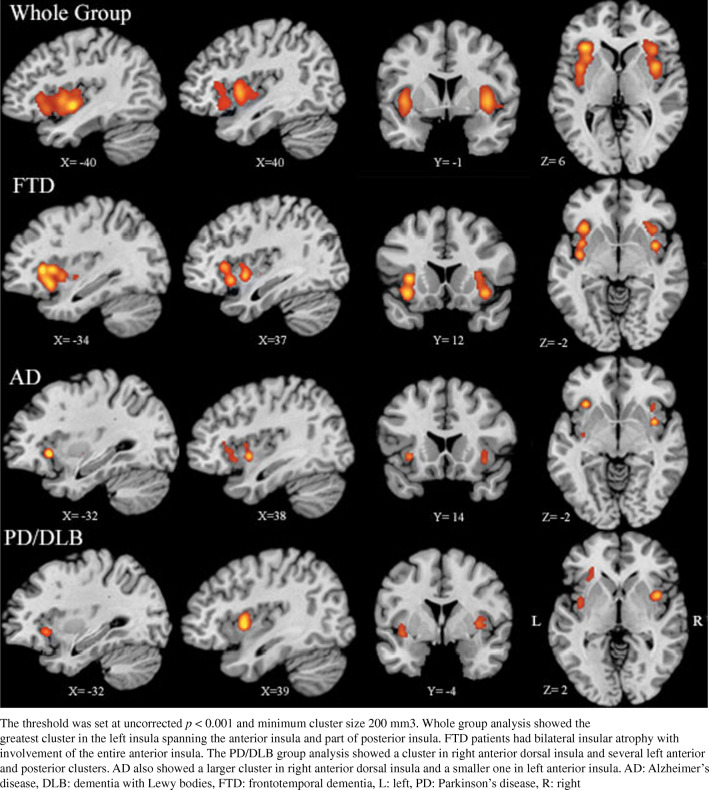
Whole group and disease-specific analysis of insular cortex atrophy in FTD, AD, and PD/DLB

Whole-group analysis of atrophy in all disease groups showed a significant convergence cluster of atrophy in the left insula (total: 14136 mm^3^). Two clusters were present in the right anterior and mid-insula combined (total:11200 mm^3^). Individual analyses were then performed for each disease group. FTD studies showed a large cluster of atrophy including most of the left insula (10,128 mm^3^) and several sub-regions in the right insula (total: 6584 mm^3^). AD studies showed involvement of the bilateral insula with a larger cluster of atrophy in the right insula (2424 mm^3^). In the PD/DLB group, a significant cluster was found in the right anterior mid-insula (2264 mm^3^) followed by two smaller clusters in ventral anterior and posterior left insula (total: 2536 mm^3^) (Fig. 3).

#### Comparison of insular sub-regional atrophy across diseases

**Fig. 4:**
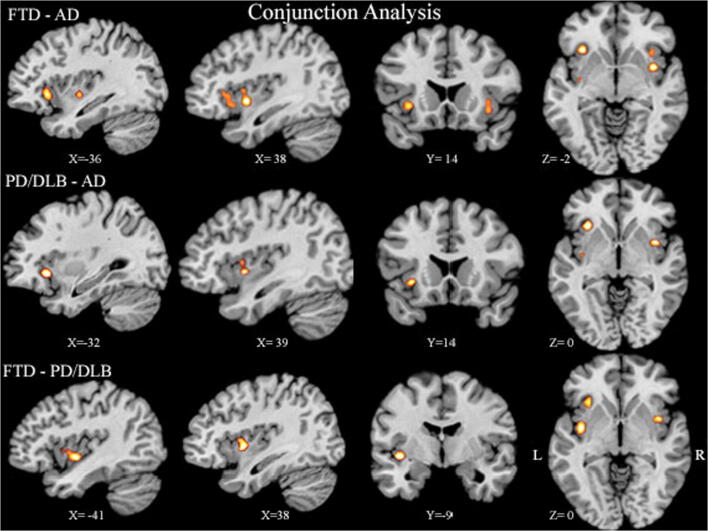
Conjunction analyses across diseases. Conjunction analyses showing overlapping insular sub-regions between FTD-AD, PD-AD, and FTD-PD are shown. The right anterior-middle dorsal and left anterior dorsal insula consistently showed atrophy across all diseases

Contrast analyses performed between FTD-AD, FTD-PD/DLB, and AD-PD/DLB did not reveal any clusters of significant difference. There were, however, overlapping clusters of insular atrophy between diseases retrieved from conjunction analyses. Insular regions with overlapping atrophy between AD and FTD showed bilateral anterior and posterior clusters. FTD-PD studies showed an area of atrophy in right mid-insula, corresponding to the posterior short gyrus of the anterior insula and anterior and mid-left insula, both microscopically corresponding to the dorsal dysgranular insula. AD-PD studies showed an overlap in the right mid-insula and two clusters in left anterior and posterior insula (Fig. 4).

#### Analysis of functional domains related to insular atrophy

**Fig. 5:**
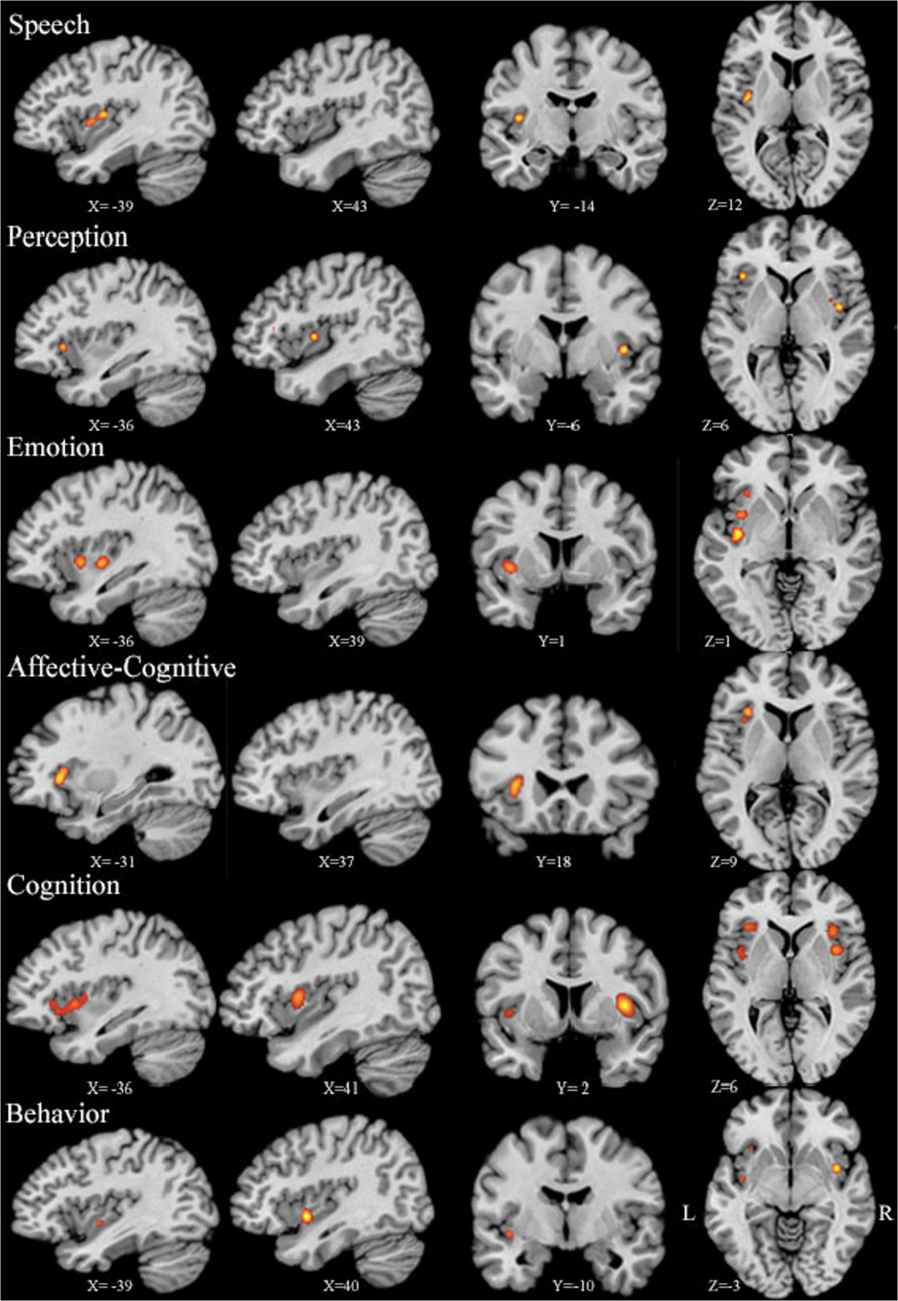
Insular atrophy and relationship with functional deficits in neurodegeneration. The threshold was set at uncorrected p < 0.001 and cluster size 200 mm^3^. The main functional domains showing deficits in relation to insular atrophy were speech, perception, emotion, affective-cognitive, cognition, and behavior. The left insula was affected in all domains except cognition where the right insula showed larger clusters in anterior and mid-dorsal insula. Coordinates are in Talairach space. L = left; R = right

Analysis of all six functional domains across all diagnoses showed hemispheric asymmetry with more prominent left insular atrophy. Left insular cortex atrophy was more pronounced in relation to speech, emotion, and affective-cognitive deficits, while the right insula showed greater atrophy in relation to cognitive impairment and perception deficits. Two clusters of atrophy extending through the left dorsal-anterior insular gyrus were found in relation to speech deficits (1288 mm^3^). In perception, a cluster was found in the right posterior dorsal insula followed by a smaller cluster in the left anterior dorsal insula (528 mm^3^ and 312 mm^3^). Affective-cognitive deficits were associated with atrophy in the left dorsal anterior insula (1128 mm^3^). Meta-analysis of emotional deficits associated with insular atrophy revealed multiple atrophic foci in anterior mid-dorsal and ventral posterior left insula (total: 2400 mm^3^). Cognitive impairment was associated with bilateral insular atrophy, comprising a larger cluster in the right anterior and mid-dorsal insula (total: 4232 mm^3^). Analysis of the behavioral functional domain revealed a large cluster in the right posterior insula and smaller clusters in left anterior and posterior ventral insula (1488 mm^3^ and 760 mm^3^). Therefore, the anterior dorsal insula was significantly atrophic in all domains except the behavioral domain which showed scattered clusters of atrophy in anterior and posterior insular cortex (Fig. 5).

## Discussion

Knowledge of the sub-regional insular cortex atrophy can provide insight into the selective vulnerability of the insular cortex and its associated clinical deficits in neurodegeneration. Based on the ALE analysis, our results illustrated greater atrophy of the left insular cortex across all diseases combined. Furthermore, the left insular cortex showed larger clusters of atrophy in FTD, compared to PD and AD which in turn showed larger convergence clusters of atrophy in the right insular cortex. Contrasting between all disease groups did not reveal any clusters of atrophy, signifying the lack of disease-specific sub-regional atrophy in the insular cortex. Conjunction analysis, assessing overlapping insular sub-regions of atrophy, showed overlapping clusters in the left anterior insula and right mid-dorsal insula across all diseases. Insular atrophy in FTD was related to emotional, speech, affective-cognitive and behavioral deficits. While atrophy in AD and PD/DLB was mostly associated with perception deficits such as psychosis as well as cognitive impairment. Left insular atrophy was also related to speech, emotional, and affective-cognitive deficits. Atrophy of the right insula, however, was associated with cognitive impairment and perception deficits. Whereas behavioral deficits were related to small clusters in bilateral insula.

### Vulnerability of the anterior dorsal insular cortex in neurodegeneration

Analysis has shown involvement of the anterior mid-dorsal insular cortex in the majority of the diseases and functional domains assessed. The anterior dorsal insula has diverse connections to limbic and cortical structures. It is preferentially connected to the orbitofrontal gyrus, olfactory cortex, entorhinal cortex, temporal pole, cingulate gyrus, and parietal cortex (Mesulam and Mufson 1982). Moreover, functional analysis of the insular sub-divisions revealed high functional diversity of this sub-region suggesting it plays an important role in integrating information necessary for cognitive functions. Due to such rich connectivity and diverse functional profile, the dorsal anterior insula could function as a brain hub (Cerliani et al. 2012; Uddin et al. 2014).

### Insular cortex atrophy-associated deficits in neurodegeneration

A variety of deficits associated with insular atrophy were found in FTD. These included deficits in understanding emotions and behaviors, speech and language deficits such as aphasia and fluency, emotional deficits such as fear and apathy, and multiple behavioral deficits including aberrant eating and disgusting behavior. Alterations in pain, temperature and physiological reactivity were also associated with insular cortex atrophy in FTD. Whereas cognitive impairment, lack of awareness and psychotic symptoms, such as delusions and visual hallucinations, were related to insular atrophy in AD as well as in PD/DLB. Several studies have also outlined the differential sub-regional involvement of the insula depending on disease stage and severity. Seeley et al. found that the anterior insula was atrophic in FTD patients with low CDR scores while those with high CDR scores and more severe cognitive impairment also had posterior insular atrophy. Similarly, Vasconcelos et al. found right anterior insular atrophy in subjects with mild AD and in correlation with disability assessment for dementia scores as well as MMSE. Although insular atrophy was commonly found in PD with MCI or dementia compared to PD with intact cognition, not all studies found a correlation with cognitive performance. This could result from the use of various cognitive tests that represent global cognitive functions rather than those related to the insular cortex such as salience processing and control of attention based on surrounding stimuli (Uddin 2015). Although it remains unclear how insular atrophy differs in early versus late disease stages, some studies highlight the role of the anterior insula in early disease stages.

### Language and speech

Language and speech, verbal agility, verbal fluency, and primary progressive aphasia showed convergence of atrophy in the left anterior mid-dorsal insula in FTD studies. This is in line with other studies showing that the left mid-dorsal insula is activated in response to speech perception tasks (Oh et al. 2014). Speech and language processing are lateralized functions depending more strongly on the left hemisphere and commonly show deficits in FTD (Hickok and Poeppel 2007; Reilly et al. 2010). Likewise, the anterior insula is a region of selective degeneration in FTD (Seeley 2010). It plays a role in the motor control of speech as well as the autonomic elements associated with it such as respiratory control (Ackermann and Riecker 2010). In a study on isolated strokes in the insular cortex, subjects that had left insular lesions exhibited aphasia while lesions in the right hemisphere were related to dysarthria (Baier et al. 2013). Nevertheless, results from various studies, on the precise involvement of the insular cortex in speech production remain inconclusive (Gasquoine 2014). Since speech is controlled bilaterally by the insula (Oh et al. 2014), while this type of aphasia typically involves the left hemisphere, it is possible that by including aphasia and speech in one analysis, our results favor the left hemisphere. Therefore, we re-analyzed the data on speech excluding aphasia but found no difference. Moreover, due to a limited sample size for speech, we analyzed aphasia and fluency together.

### Emotional and affective-cognitive deficits in neurodegeneration

Furthermore, emotional deficits including apathy, fear, agitation, and happiness were associated with convergence of atrophy in the left ventral posterior & bilateral mid-dorsal insula. Analysis of apathy only showed similar clusters of atrophy. Apathy is defined by lack of emotions, motivation, and interest and constitutes a common finding in patients with neuropsychiatric disorders. In AD and PD patients, up to 70% and 90% of patients suffer from apathy, respectively (Cummings et al. 2015). Apathy was also found to predict progression from normal cognition to MCI and from MCI to dementia in AD patients (Guercio et al. 2015). Apathy is a complex disorder including an emotional component related to the reward of completing an action as well as a cognitive component for the execution of an action manifesting as lack of goal-directed behavior (Boublay et al. 2016; Kos et al. 2017). The anterior insular cortex also represents a primary structure involved in salience processing which, by detecting salient stimuli and directing attention, provides a motivational context to external stimuli (Menon and Uddin 2010). This could explain the role of the insular cortex in apathy.

Similarly, complex functions related to enhancement of memory through emotions, recognition of emotions, and empathy were particularly affected in FTD. The analysis of this affective-cognitive category revealed a cluster in the left anterior dorsal region. Deficits in recognizing emotions or behaviors occur in several neurodegenerative diseases including FTD, PD, and AD. These functions are important for the development of proper interpersonal skills and constitute multiple underlying functions such as perception and social judgement (Goodkind et al. 2015). Degeneration of the anterior dorsal insula could thus contribute to the processing of functions involving multiple domains due to its role in emotional and cognitive processing.

### Insular atrophy behind hallucinations and delusions in AD and PD

Perception deficits including hallucinations and delusions, which both represent psychotic symptoms, were present in AD, PD and DLB patients. Analysis showed a cluster of atrophy in the right dorsal posterior insula and a smaller cluster in the left anterior dorsal insula. Hallucinations are perceptions generated by the mind that exist without the presence of external stimuli, while delusions are abnormal and false beliefs ranging from prosecutory to content specific (Padilla and Mendez 2016). Hallucinations and delusions are common in several neurodegenerative disorders and can have significant burdening effects on patients due to their intrusive nature (Burghaus et al. 2012). The insular cortex functions in integrating internal information and external sensory inputs (Mesulam and Mufson 1982). The anterior part of the insula also plays a role in self-awareness and attention (Craig 2009). Dysfunction of attention networks including the insular cortex could thus contribute to visual hallucinations (Shine et al. 2014). Last, the behavioral deficits, which were most common in FTD, included aberrant eating and disgust behaviors. This domain was associated with atrophy of multiple sub-regions of bilateral insula yet it remains unclear how insular atrophy could contribute to these deficits.

### Limitations and future perspectives

In this meta-analysis, we only studied the role of the insular cortex in disease. Since higher functions such as cognition and emotion are the result of an interplay between various regions and networks, focusing only on the insular cortex is a limitation of this study. Nevertheless, studies have identified the role of various brain regions in networks and their subsequent involvement in behavior but information on the contribution of individual brain regions to function has been lacking (Genon et al. 2018). Similarly, the insular cortex has been implicated to play a central role in the salience network and is identified as a hub affected in various diseases and behaviors, yet it has been unclear precisely what contribution it has to the deficits associated with neurodegeneration and whether vulnerability to degeneration is indeed variable across its sub-divisions. Moreover, the focus of this review is only on VBM studies, other neuroimaging modalities were not included. Although VBM is an automated method to assess volume changes across conditions, it is affected by variation in methodologies such as scanner and image quality, pre-processing type, and statistical analysis (Scarpazza et al. 2015). Other limitations are related to technical differences across studies such as smoothing kernel sizes, thresholds, significance levels, and method used for correction of multiple comparison (Whitwell 2009). On the other hand, VBM is an unbiased method used to detect subtle brain changes. It has been useful in identifying regional differences in grey and white matter, common in neurodegenerative diseases, and has shown regional differences in atrophy across diseases as well as relationships with other clinical deficits (Whitwell and Jack Jr 2005; Whitwell and Josephs 2007). ALE is a peak-based method of meta-analysis which relies on pooling peak coordinates rather than using raw maps and thus may yield less accurate results. Furthermore, in this meta-analysis we used an uncorrected *p* < 0.001 which, despite its conservative value, may have led to false positive results. As previously mentioned, while cluster analysis may have been more sensitive, its use is debatable for VBM studies. Similarly, FDR and FWE approaches are conservative and would have yielded limited results. Since we used a region-of-interest approach by focusing on the insular cortex only, we wanted to be maximally sensitive to small yet meaningful effects. Hence, we opted to use a more liberal methodological approach, uncorrected p, for this meta-analysis. Overall, this study provides a comprehensive summary and quantitative analysis of the relationship between insular cortex sub-regional atrophy across four common neurodegenerative diseases and their corresponding cognitive and neuropsychiatric deficits. Due to the heterogeneity of the insular cortex sub-regions, both structurally and functionally, it is imperative to understand how these regions contribute differentially to neurodegenerative diseases. Even though neuroimaging studies have shed much light onto the role of the insular cortex in disease, several limitations exist. Namkung et al. have recently proposed translational and back-translational approaches to unravel the complex role of the insular cortex. To study causal relationships, Granger causality analysis as well as further computational models and statistical efforts for neuroimage processing along with non-invasive imaging modalities could be used. Furthermore, animal studies could shed some light on the contribution of various neural circuitry elements to behavior. Therefore, to obtain a comprehensive understanding of the complex physiological role of the insular cortex as well as contribution to disease, more research at molecular and cellular levels combined with advanced neuroimaging analyses would be needed (Namkung et al. 2017).

## Conclusions

Our meta-analysis showed that atrophy of the insular sub-regions is not disease-specific. Yet, the anterior and middle dorsal insula were atrophic in all included neurodegenerative diseases. The anterior insular cortex, particularly the dorsal insula comprising of a diverse array of limbic and cortical connections, contributed to a broad range of deficits in all neurodegenerative diseases. Our study illustrates the presence of specific patterns of atrophy in the anterior dorsal insula in neurodegeneration which are associated with deficits in speech, emotional, affective-cognitive, perception and cognition. These patterns of insular sub-regional atrophy could aid in understanding clinical heterogeneity in neurodegenerative diseases and provide potentially beneficial information for future biomarker studies.

## Electronic supplementary material

ESM 1 (DOCX 15 kb)

## Abbreviations

AD: Alzheimer’s disease
ADAS-TES: Alzheimer’s disease assessment scale- Total error score
bvFTD: behavioral variant frontotemporal dementia
CDR: Clinical Dementia Rating
dAI: Dorsal anterior insula
DLB: Dementia with Lewy bodies
FM: family member
FTD: Frontotemporal dementia
FWE: Family-wise error
HC: Healthy controls
LPA: Logopenic aphasia
MCI: mild cognitive impairment
MCI: Mild cognitive impairment
nfvPPA: non-fluent variant primary progressive aphasia
PD: Parkinson’s disease
PDD: Parkinson’s disease dementia
PI: Posterior insula
PNFA: Progessive non-fluent aphasia
PSP: Progressive supranuclear palsy
rtFTD: right temporal variant FTD
SD: Semantic dementia
Soc/Exec: Social/ executive disorder FTD
SPGI: Superior precentral region of the dorsal anterior insula
TOM: Theory of mind
vAI: Ventral anterior insula
