# Supplementary material for: Differential insular cortex sub-regional atrophy in neurodegenerative diseases: a systematic review and meta-analysis
Source: Brain Imaging Behav. 2019 Apr 23;14(6):2799–816. doi: 10.1007/s11682-019-00099-3 (PMC7648006; doi:10.1007/s11682-019-00099-3)
Supplement: Supplementary file 1 — (DOCX 15 kb) [file 11682_2019_99_MOESM1_ESM.docx]

Supplementary

**Table I. Functional domains and corresponding measurement scales**

| **Study** | **Function** | **Patient Group** | **Scale** |
| --- | --- | --- | --- |
| **Speech** | | | |
| (Mandelli et al., 2016) | Verbal agility | FTD | Western aphasia battery & motor speech evaluation |
| (Hu et al.,2010) | Aphasia | FTD | Clinical diagnosis |
| (Ash et al., 2009) | Speech fluency | FTD | Story narration |
| **Perception** | | | |
| (Ting et al., 2015) | Delusions | AD | NPI-Q |
| (Blanc et al., 2014) | Delusions | AD | NPI |
| (Gama et al., 2014) | Hallucinations | PD | Visual analog scale |
| (Shine et al., 2014) | Hallucinations | PD | Bistable percept paradigm |
| (Nakaaki et al., 2013) | Delusions | AD | NPI |
| **Affective- Cognitive** | | | |
| (Woolley et al., 2015) | Recognition of Disgust | FTD | TASIT-EET Disgust sub-score |
| (Kumfor et al., 2014) | Emotional enhancement of memory | FTD | Emotional memory task |
| (Couto et al., 2013) | Facial Emotion Recognition | FTD | cartoon |
| (Kumfor et al., 2013) | Facial Emotion Recognition | FTD | Ekman 60   Ekman Caricatures tasks. |
| (Omar et al., 2011) | Music Emotion Recognition | FTD | Ekman |
| (Dermody et al., 2016) | Empathy | FTD | Interpersonal Reactivity Index |
| **Emotion** | | | |
| (Alzahrani et al., 2016) | Apathy | PD | NPI and DSM-IV-TR |
| (Sturm et al., 2015) | Happiness- Emotional Reactivity | FTD | Laboratory |
| (Hu et al., 2015) | Agitation | AD | NPI-Q |
| (Stanton et al., 2013) | Apathy | AD | AES and NPI |
| (Eslinger et al., 2012) | Apathy | FTD | AES |
| (Reijnders et al., 2010) | Apathy | PD | AES, NPI, LARS |
| (Hoefer et al., 2008) | Fear conditioning | FTD | Fear conditioning paradigm |
| **Cognition** | | | |
| (Heitz et al., 2016) | TOM | DLB | FPR, RME, EFER |
| (Chen et al., 2016) | Global cognitive abilities | PD | MOCA, MMSE |
| (Mak et al., 2014) | Executive functions and attention | PD | FAB, digit span, colour trails 2 and time taken on a Maze test |
| (Lee et al., 2014) | Executive functions | PD | Contrasting programme, go-no-go test, Luria loop, phonemic and semantic Controlled Oral Word Association Test, and Stroop test |
| (Shany-Ur et al., 2014) | Self-awareness | AD & FTD | Patient Competency Rating Scale |
| (Lee et al., 2013) | Cognition | PD | MMSE |
| (Couto et al., 2013) | Theory of mind | FTD | RME |
| (Farrow et al., 2007) | Cognition | AD | ADAS-TES |
| **Behavior** | | | |
| (O'Callaghan et al., 2016) | Social normative behavior | FTD | Ultimatum Game |
| (Cerasa et al., 2014) | Pathological gambling | PD | G-SAS, BIS-11 |
| (Woolley et al., 2015) | Disgust behavior | FTD | Disgust scale, clinical |
| (Fletcher et al., 2015) | Auditory hedonia | FTD | Questionnaire |
| (Whitwell et al., 2007) | Feeding behavior | FTD | MOUSEPAD |
| (Woolley et al., 2007) | Feeding behavior | FTD | Eating experiment |
| (Rosen et al., 2005) | Behavior abnormalities | FTD | NPI |

**Supplementary Table I. Functional domains and measurement scales.** All 6 functional categories and corresponding functions studied in the meta-analysis are shown. **AES:** apathy evaluation scale, **ADAS-TES**: Alzheimer’s disease assessment scale- total error score, **BIS-11**: Barratt impulsivity scale, **CDR**: Clinical dementia rating, **EFER**: Ekman facial emotion recognition test, **FAB**: frontal assessment battery, **FPR**: Faux Pas Recognition test, **G-SAS**: gambling symptom assessment scale, **LARS**: Lille apathy rating scale, **MOUSEPAD**: Manchester and Oxford Universities Scale for the Psychopathological Assessment of Dementia, **NPI**: Neuropsychiatric inventory questionnaire, **RME**: Reading the Mind in the Eyes test, **TASIT-EET:** Emotion Evaluation subtest of The Awareness of Social Inference Test, **TOM**: theory of mind
